# An effective combination of whole-exome sequencing and runs of homozygosity for the diagnosis of primary ciliary dyskinesia in consanguineous families

**DOI:** 10.1038/s41598-017-08510-z

**Published:** 2017-08-11

**Authors:** Ting Guo, Zhi-Ping Tan, Hua-Mei Chen, Dong-yuan Zheng, Lv liu, Xin-Gang Huang, Ping Chen, Hong Luo, Yi-Feng Yang

**Affiliations:** 1 Department of Respiratory Medicine, the Second Xiangya Hospital, Central South University, Changsha, Hunan 410011 China; 2 Research Unit of Respiratory Disease, Central South University, Changsha, Hunan 410011 China; 3 Diagnosis and Treatment Center of Respiratory Disease, Central South University, Changsha, Hunan 410011 China; 4 Central South University Center for Clinical Gene Diagnosis and Treatment, the Second Xiangya Hospital, Central South University, Changsha, Hunan 410011 China; 5 Department of Cardiovascular Surgery, the Second Xiangya Hospital, Central South University, Changsha, Hunan 410011 China; 6 Department of Respiratory Medicine, Chang Sha Central Hospital, Changsha, Hunan 410011 China

## Abstract

Primary ciliary dyskinesia (PCD) is clinically characterized by neonatal respiratory distress, chronic sinusitis, bronchiectasis and infertility, and situs inversus in 50% of the patients. PCD is a result of mutations in genes encoding proteins involved in ciliary function, and is primarily inherited in an autosomal recessive fashion. Diagnosis of PCD is often a challenging task due to its high clinical and genetic heterogeneities. In the present study, we attempted to use whole-exome sequencing (WES) combined with runs of homozygosity (ROH) approaches to identify the genetic defects in four Chinese consanguineous families with clinical PCD. We successfully identified three recently acknowledged PCD genes: *DYX1C1*, *CCNO* and *ARMC4*, and one well-characterized PCD gene, *DNAI1*. Our study provides compelling evidence that WES in combination with ROH analysis is an efficient diagnostic tool for identifying genetic causes of PCD in consanguineous families. Furthermore, our work expands the genetic mutation spectrum in PCD, and provides the additional tools to better serve the counseling of the families with PCD.

## Introduction

Primary ciliary dyskinesia (PCD; MIM: 244400) is a clinically heterogeneous disorder due to impairment of ciliary function and has an estimated prevalence of approximately 1 in 15,000–20,000 individuals^1^. Ineffective cilium movement of cells lining the upper and lower respiratory tracts may result in limited mucus clearance, leading to neonatal respiratory distress, rhinitis, sinusitis, rhinorrhea, chronic cough, recurrent respiratory infections and bronchiectasis. Male infertility may occur as functional cilia are required for proper sperm flagella function. Approximately 50% of the PCD patients present with situs inversus due to the cilium dysfunction during early embryonic development^1, 2^.

Currently, there is no “gold standard” reference test for PCD. The traditional diagnosis of PCD largely depends on the careful evaluation of clinical features, ciliary function, ultrastructural defects, nasal nitric oxide (nNO) levels, and genetic analysis^2^. However, some of these diagnostic approaches are not easily accessible and require specialized expertise. Some tests are challenging due to the heterogeneity of the disease, including atypical or overlapping phenotypes, which could lead to incorrect clinical diagnosis and suboptimal treatment.

PCD is primarily a rare autosomal recessive disorder caused by mutations in genes encoding proteins involved in cilium function^1^. As a Mendelian genetic disorder, PCD can be identified through genetic analysis. Since the first identified disease gene *DNAI1* in PCD patients with immotile respiratory cilia in 1999^3^, remarkable efforts have been made to define the genetic cause of PCD. So far, genetic mutations in over 40 genes account for an estimated 65% of individuals^1, 4–10^. However, approximately 35% of PCD individuals remain genetic elusive^11^, highlighting the genetic heterogeneity and challenges of using conventional targeted gene panels based on the currently known PCD-associated genes. Recently, WES in combination with multiple strategies, including runs of homozygosity (ROH)^12, 13^, linkage analysis^14^, and filtering of reported disease-related genes^15–18^, have shown tremendous potentials for genetic diagnosis and for identification of novel variants. In some cases, these strategies appear to be the only way for achieving a precise diagnosis^19^.

The prevalence of PCD has a higher rate in consanguineous families^20^ and has been occasionally reported in China. We hypothesize that the consanguineous families with PCD provide a great opportunity to recover novel causative PCD gene(s). In the present study, we examine the clinical diagnosis of PCD with WES in combination with ROH. Six PCD patients from four consanguineous families were analyzed using WES, and a total of four PCD causative genes, *CCNO*, *DYX1C1*, *ARMC4* and *DNAI1* were identified in these consanguineous families.

## Materials and Methods

### Patients and subjects

**Figure 1:**
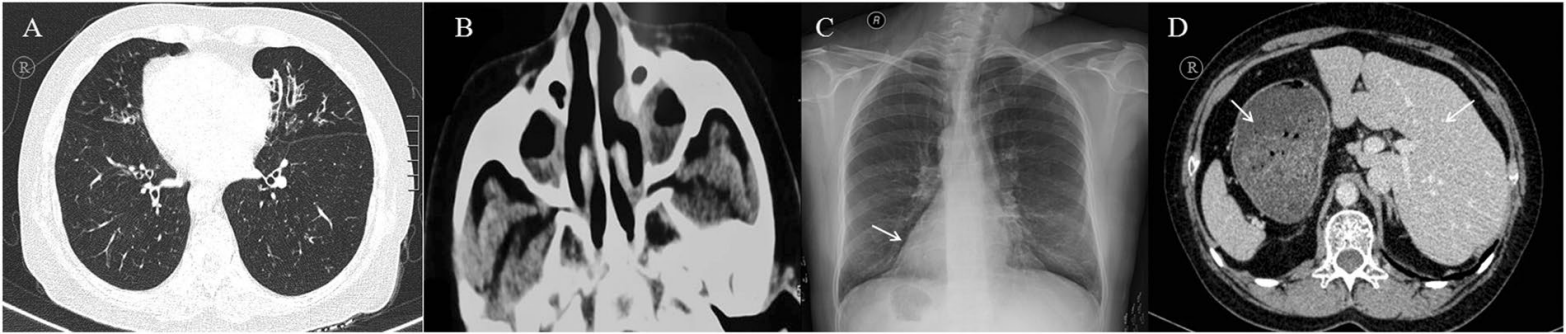
Clinical features of PCD in the consanguineous families. The patient (P6 from family 4) presented the classical clinical features of PCD. (**A**) The chest computed tomography (CT) scan showed bronchiectasis. (**B**) The paranasal sinus CT scan of the patient showed apparent rhinosinusitis. Typical situs inversus is shown in C, D. CT images of the remaining patients are shown in Supplementary Figure 1.

The study protocol was approved by the Review Board of the Second Xiangya Hospital of Central South University in China. Informed consent was obtained from all subjects. All experiments were performed in accordance with the relevant guidelines and regulations. Four consanguineous families with PCD were recruited, in which one individual was affected in families 1 and 4, and two individuals were affected in families 2 and 3. The medical records of the subjects, including pulmonary, paranasal sinus, abdominal computed tomography (CT) (Fig. 1 and Supplementary Figure 1), nNO level^21^, and transmission electron microscopy (TEM) findings (Supplementary Figure 2) were documented and reviewed.

### WES and filtering

**Figure 2:**
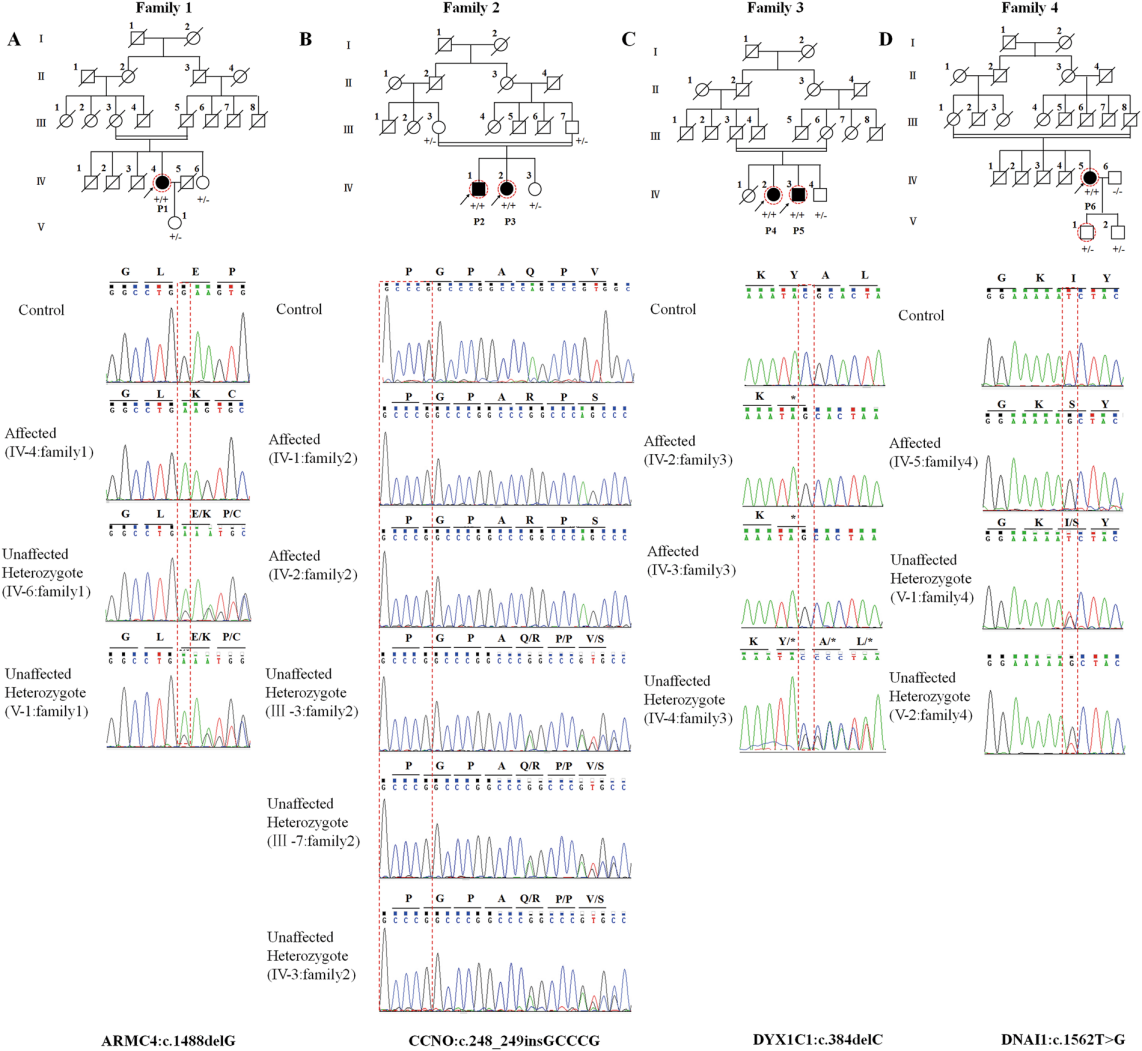
Pedigree of the families and the Sanger DNA sequencing chromatogram of the patients. Roman numerals refer to generations, and individuals within a generation are numbered from left to right. Black circles/squares are affected, and white circles/squares are unaffected. Arrows indicate the probands. P1-P6 are the probands (P1 refers to IV-4 in family 1; P2 and P3 refer to IV-1 and IV-2 in family 2, respectively; P4 and P5 refer to IV-2 and IV-3 in family 3, respectively; and P6 refers to IV-5 in family 4). Seven large circles (in red) represent the 7 individuals who underwent WES. The Sanger sequencing chromatogram of the affected patient, unaffected carrier, and control is shown under each pedigree. The genotype is indicated in the pedigree charts:+/+, affected patient (homozygous mutation);+/−, unaffected carrier (heterozygous mutation); and −/−, unaffected control.

Blood samples (3–5 ml) were obtained from the affected probands and their family members (Fig. 2). Genomic DNA was extracted using the DNeasy Blood & Tissue Kit (Qiagen, Valencia, CA). Whole-exome capture and high-throughput sequencing (HTS) were performed by the Novogene Bioinformatics Institute (Beijing, China). Briefly, whole exomes were captured using the Agilent SureSelect Human All ExonV5 Kit (Agilent, California, USA) and sequenced on the Illumina HiSeq. 2500 platform. The sequencing reads were aligned to the human reference genome (UCSC hg19), and the details of the sequencing data are provided in Supplementary Table S1. Single nucleotide variants (SNVs) and short insertions and deletions (InDels) were filtered as follows: (i) Variants within intergenic, intronic, and UTR regions and synonymous mutations were excluded from subsequent analyses. (ii) High-frequency (minor allele frequency >0.01) polymorphisms found in the 1000 Genomes Project, ESP6500, Exac, and Novogene Bioinformatics Institute in-house exomeSeq databases were excluded. (iii) Based on the principle that the identification of a new disease-causing gene should exclude known causal genes^17^, variants in the 40 PCD-related genes (Supplementary Table S2) were selected. This approach prevented the omission of any compound heterozygotes of known genes. (iv) Due to the examination of consanguineous families, ROH^12, 13^ analysis was performed. ROH analysis is an important approach because it can effectively exclude false-positive variants in the case of a large deletion on the other allele. The detailed steps of filtering are shown in Supplementary Figure 3.

### Bioinformatic analysis

The effect of the identified variants on protein function was predicted using bioinformatic programs (Sift, Polyphen-2, MutationTaster, PROVEAN, LRT and MutationAssessor), and potential effects on protein structure were determined using the Swiss Model tool. To evaluate the degree of conservation, alignment of the amino acid sequences of the targeted genes from various species was obtained from MutationTaster (http://www.mutationtaster.org), and domain analyses were performed (http://www.ncbi.nlm.nih.gov/Structure/cdd/wrpsb.cgi).

### Mutation validation and co-segregation analysis

Sanger sequencing was used to validate the candidate variants identified by WES, and segregation analyses were performed in the family members. Primer pairs were designed using an online tool (PrimerQuest, IDT), and the sequences of the primers are listed in Supplementary Table S3.

## Results

### Clinical features

Six PCD patients (P1–6) (Table 1) from four consanguineous families (families 1 to 4) (full pedigrees are shown in Fig. 2) were identified. P1 refers to IV-4 in family 1; P2 and P3 refer to IV-1 and IV-2 in family 2, respectively; P4 and P5 refer to IV-2 and IV-3 respectively in family 3; and P6 refers to IV-5 in family 4 (see Fig. 2). The parents of all probands are first cousins, and the inbreeding coefficient (F) is 1/16 for each family. All patients in these families presented with typical PCD manifestations, including recurrent upper and lower respiratory infections from childhood, bronchiectasis, chronic rhinosinusitis, left-right laterality, reduced nNO or infertility. The specific clinical manifestations of each patient are shown in Table 1.

**Table 1:** Summary of the clinical features of PCD.

Characteristics	P1 (family1:IV-4)	P2 (family2:IV-1)	P3 (family2: IV-2)	P4 (family3: IV-2)	P5 (family3: IV-3)	P6 (family4: IV-5)
Sex	Female	Male	Female	Female	Male	Female
Age	60 y	29 y	23 y	43 y	41 y	52 y
Onset	Childhood	Childhood	Childhood	Childhood	Childhood	Childhood
Consanguinity	Yes	Yes	Yes	Yes	Yes	Yes
Inbreeding coefficient	1/16	1/16	1/16	1/16	1/16	1/16
CT	Bronchiectasis Rhinosinusitis Visceral inversion	Bronchiectasis Rhinosinusitis	Bronchiectasis Rhinosinusitis	Bronchiectasis Rhinosinusitis Visceral inversion	Bronchiectasis Rhinosinusitis Visceral inversion	Bronchiectasis Rhinosinusitis Visceral inversion
Fertility problems	No	Unknown	Unknown	Yes	Yes	No
Smell problems	No	No	No	Yes	Yes	No
Hearing problems	No	No	No	No	No	No
Comorbidities	Asthma	No	No	TGA,ASD,PAH	No	Asthma
TEM	NA	NA	NA	NA	NA	Equivocal
PFT						
FEV1%	32%	33%	49%	44%	NA	37.2%
FEV1/FVC	40.21%	54.73%	50%	60%	NA	36.37%
PaO2	NA	78 mmHg	82 mmHg	80 mmHg	NA	NA
Nasal NO	23.6 ppb	3.3 ppb	31 ppb	2.3 ppb	8.3 ppb	16.1 ppb

TGA, corrected transposition of great arteries; ASD, atrial septal defect; PAH, pulmonary arterial hypertension; TEM, transmission electron microscopy; PFT, pulmonary function test; FEV1, forced expiratory volume in 1 second; FVC, forced vital capacity; Nasal NO, nasal nitric oxide measurement; NA, not available.

### Genetic analysis

WES was performed on seven individuals (Fig. 2). Sequence read of 7.88, 5.65, 4.9, 8.63, 8.23, 5.46 and 6.48 Gbp were generated, with a depth of more than 50 × for each individual, and more than 99% of the targeted bases were covered sufficiently to pass our thresholds for SNVs and InDels (Supplementary Table S1). Exonic InDels, non-synonymous variants, and nonsense and splice-site SNVs were further filtered. After the discovery of rare variations (minor allele frequency <1%), selection of variants in the 40 PCD-related genes (Supplementary Table S2) was performed. The coverage of the whole exome by the exome capture kit was approximately 99.7–99.8%, and the coverage of the 40 known PCD genes was greater than 99% (Supplementary Table S4). No compound heterozygotes of known PCD-related genes were identified in these patients. Interestingly, only one homozygous mutation in a known PCD gene was identified in each family (c.1488delG of *ARMC4* in family 1, c.248_249insGCCCG of *CCNO* in family 2, c.384delC of *DYX1C1* in family 3, and c.1562T > G of *DNAI1* in family 4). All four variants were validated via Sanger sequencing (the PCR primers are listed in Supplementary Table S3). The sequences of these genes from each of these four families were determined, and the variations were found to co-segregate with the disease in each family (Fig. 2).The frequencies of the identified variants in the public databases (Exac, ClinVar, NHLBI exome databases, dbSNP138, 1000 Genomes Project) are shown in Supplementary Table S5.

Among the four apparently homozygous variants identified in our study, three were nonsense/frameshift mutations and one was missense mutation (Table 2). To exclude the possibility of a large deletion of the other homologous allele, we performed ROH analysis^12, 13^. In accordance with the autosomal recessive pattern of inheritance, our data from ROH analysis confirmed the homozygous conditions of these four mutations in all four PCD families.

**Table 2:** Functional prediction and conservation analysis for the cosegregating mutation

Family	Gene	NM	Base change	AAchange	MutationTaster	PROVEAN	SIFT	Polyphen2	LRT	MutationAssessor	CADD	GERP++	phylop100way
1	*ARMC4*	018076	1488delG	E497Kfs*3	Disease-causing	Deleterious	NA	NA	NA	NA	NA	NA	NA
2	*CCNO*	021147	248_249insGCCCG	Q88Rfs*8	Disease-causing	Deleterious	NA	NA	NA	NA	NA	NA	NA
3	*DYX1C1*	130810	384delC	Y128*	Disease-causing	Deleterious	NA	NA	NA	NA	NA	NA	NA
4	*DNAI1*	012144	T1562G	I521S	Probably damaging	Deleterious	Damaging	Disease-causing	Deleterious	Medium	23	5.95	7.002

CADD, a value > 15 indicates deleterious; GERP++, a score > 5 indicates highly conserved; phylop100way, the higher the score, the more conserved the site AA, amino acid NA, not available.

Consistent with the loss of function mechanisms of the known PCD-linked genes, the mutations identified in *ARMC4*, *CCNO*, and *DYX1C1* in three of the families are either deletions or insertions, leading to loss of function by causing truncation of the encoded proteins. However, c.1562T > G (I521S) of *DNAI1*, found in family 4, was a missense mutation that alters a highly conserved residue (Supplementary Figure 4). The I521S mutation was predicted to be damaging according to SIFT, Polyphen2, MutationTaster, PROVEAN, LRT and MutationAssessor. The GERP score was 5.95 (prediction: highly conserved); the CADD value was 23 (prediction: deleterious); and the phylop100way value was 7.002 (prediction: conserved) (Table 2). Domain analysis suggested all of the variants were located in key domains that are essential for function of cilia (Supplementary Figures 4 and 5).

**Figure 3:**
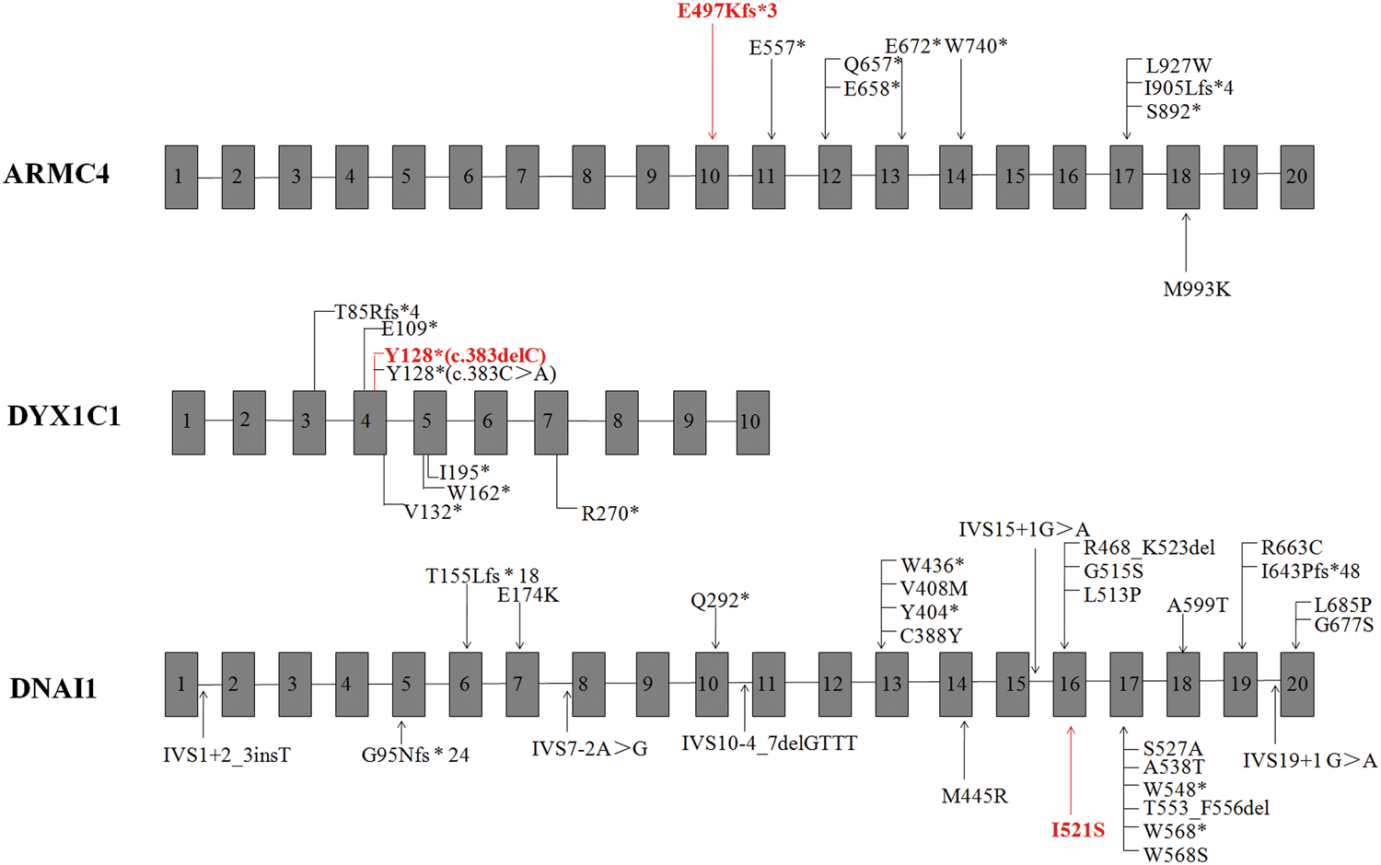
Schematic drawing of *ARMC4*, *DYX1C1*, and *DNAI1*, in which the positions of all identified mutations are indicated. Novel mutations that were identified in the present study are indicated in red.

The identified *CCNO* variant (Table 2) (c.248_249insGCCCG/p.Q88Rfs*8)^22^ was previously reported to be linked to PCD. The remaining three mutations are novel (Fig. 3). None of these three mutations were found in our in-house 200 controls and other public databases, including ExAC.

## Discussion

In the present study, WES combined with ROH was employed to determine the causative genes in four consanguineous families with PCD. Four distinct homozygous mutations were identified in these families. Among them, three of the variants are either deletions or insertions leading to frameshift (Table 2). Our findings are consistent with previous studies that the majority of mutations reported in PCD patients are null alleles (nonsense or frameshift variants)^23^. A novel missense variant (c.1562T > G/p.I521S) in *DNAI1* was identified in Family 4 and was predicted to be disease-causing variant by different in silico prediction methods. This variant site (I521) located in the WD40 domain, a key domain for the assembly and maintenance of cilia, strongly indicates the *DNAI1* variant is causative in this case. Cumulatively, a total of 12 missense mutations (out of the 27, Fig. 3) have been reported in *DNAI1*. Among these, several missense mutations closely flanking I521, including L513P, G515S, S527A and A538T^24–27^ have previously been identified in PCD, suggesting missense mutations represent a significant fraction of PCD-linked mutations in *DNAI1*.

The identification of ultrastructural defects through transmission electron microscopy (TEM) has been previously considered a “gold standard” diagnostic test for PCD^28^. However, it was challenged by recent findings^29–32^, due to its limited sensitivity. Approximately 30% of patients with genetically verified PCD exhibit a normal ciliary ultrastructure^29^. Of note, defective cilia have also been found in 3 to 10% of healthy individuals^30^. In our study, the affected individuals present some of the typical PCD manifestations such as: recurrent respiratory infections, bronchiectasis, left-right laterality and low nasal nitric oxide (nNO) levels (3.3–31 ppb in contrast to normal values >100 ppb) (Table 1). However, the TEM results of the only obtained ciliated sample from P6 are not conclusive (Supplementary Figure 2). In consistent with European guidelines for PCD diagnosis, we further employed WES to investigate these consanguineous families. In the absence of TEM evidence, our strategy emphasizes a role of genetic testing in the PCD diagnostic, especially in cases with difficulties using other diagnostic approaches (e.g., HSVA, TEM or IF)^33^.

In the present study, the filtering of the 40 PCD genes was performed prior to ROH analysis, as this order allowed the identification of compound heterozygotes in known PCD genes. Copy number variations are prevalent in many autosomal recessive diseases^17^. However, the detection of copy number variations using WES data remains challenging^34^. ROH analysis presents the advantages of determining the exact nature of mutations in this aspect. Our findings are consistent with a recent report^35^ in which WES combined with ROH was shown to be a superior choice for genetic studies in consanguineous families. As our study was primarily aimed at detecting PCD mutations, we did not actively search for “the incidental findings” that are recently recommended by the American College of Medical Genetics and Genomics^36^.

To date, a total of 40 known PCD-causing genes have been reported (Supplementary Table S2). Among these genes, *ARMC4* (MIM: 615408) encodes a protein required in a late step for proper targeting and anchoring of outer dynein arms (ciliary structure proteins)^37^. Thus far, 9 different *ARMC4* mutations (2 missense mutants, 6 truncated mutants, and 1 frameshift mutant) have been identified in PCD patients (Fig. 3), with most of these mutations being found in exons 17 and 12. The frameshift mutation *ARMC4* (c.1488delG/p.E497Kfs*3) in our study is located in the ARM-repeat unit (ARM) domain (Supplementary Figure 4) and is predicted to cause premature protein truncation, resulting in the assembly defect of the outer dynein arm.

The *DYX1C1* gene (MIM: 608706) was another gene reported to be involved in PCD^38^. *DYX1C1* encodes a 420-amino acid protein with 3 tetratricopeptide repeat (TPR) domains that are thought to be protein interaction modules. DYX1C1 is a cytoplasmic axonemal dynein assembly factor, acting together with DNAAF2 at an early step in the cytoplasmic assembly of the inner and outer dynein arms^38^. To date, seven pathogenic variants of *DYX1C1* have been reported^38^. Our finding that a single-base deletion of C at position 384 (c.384delC) results in p.Y128* is consistent with the previous report by Tarkar *et al*.^38^.

The pathogenetic variant (c.248_249insGCCCG/p.Q88Rfs*8) identified in *CCNO* (MIM: 607752) was previously reported^22^. Due to the lack of situs inversus phenotype, the patients in family 2 were initially diagnosed with common pulmonary infection and bronchiectasis. Additional examinations revealed that both siblings exhibited chronic sinusitis symptoms, which was further confirmed via computerized tomography (CT) scan of the paranasal sinuses. Genetic analysis identified only one candidate variant *CCNO* (c.248_249insGCCCG/p.Q88Rfs*8). *CCNO* promotes mother centriole amplification and maturation in preparation for apical docking and ciliogenesis. The phenotype of our patients was consistent with the observation in previous study^22^. Retrospective study revealed a delayed diagnosis in our patient due to the atypical clinical PCD features (without situs inversus)^1^. This highlights the advantage of early genetic analysis with WES to obtain an efficient diagnosis and management of PCD patients in cases of consanguinity.

In summary, using a combination of WES, ROH and bioinformatics analyses, three novel homozygous variants, including *ARMC4* (c.1488delG/p.E497Kfs*3), *DYX1C1* (c.384delC/p.Y128*) and *DNAI1* (c.1562T > G/p.I521S), and one previously reported variant, *CCNO* (c.248_249insGCCCG/p.Q88Rfs*8), were identified in four PCD families. The identification of these variants contributes to the growing number of candidate mutations associated with PCD and might benefit future genetic counseling. Our data have also demonstrated a great value of WES combined with ROH in PCD diagnosis in consanguineous families, especially when the other diagnostic tests fail to be conclusive.

## Electronic supplementary material

Supplementary
