# Supplementary material for: An effective combination of whole-exome sequencing and runs of homozygosity for the diagnosis of primary ciliary dyskinesia in consanguineous families
Source: Sci Rep. 2017 Aug 11;7:7905. doi: 10.1038/s41598-017-08510-z (PMC5554225; doi:10.1038/s41598-017-08510-z)
Supplement: Supplementary file 1 — Supplementary [file 41598_2017_8510_MOESM1_ESM.pdf]

An effective combination of whole-exome sequencing and runs of homozygosity for the diagnosis of primary ciliary dyskinesia in consanguineous families

Ting Guo[1,2,3#], Zhi-Ping Tan[4,5#], Hua-Mei Chen[6], Dong-yuan Zheng[1,2,3], Lv liu[1,2,3], Xin-Gang Huang[1,2,3], Ping Chen[1.2.3], Hong Luo[1,2,3 *] and Yi-Feng Yang[4, 5*]

1. Department of Respiratory Medicine, the Second Xiangya Hospital , Central South University, Changsha, Hunan 410011, China.
2. Research Unit of Respiratory Disease, Central South University, Changsha, Hunan 410011, China.
3. Diagnosis and Treatment Center of Respiratory Disease, Central South University, Changsha, Hunan 410011, China.
4. Clinical Center for Gene Diagnosis and Therapy of State Key Laboratory of Medical Genetics, the Second Xiangya Hospital ,Central South University, Changsha, Hunan 410011, China.
5. Department of Cardiovascular Surgery, the Second Xiangya Hospital, Central South University, Changsha, Hunan 410011, China
6. Department of Respiratory Medicine, Chang Sha Central Hospital, Changsha, Hunan 410011, China.

[#]Ting Guo and Zhi-Ping Tan contributed equally to this article.

* Correspondence to: Hong Luo (Luohong1003@163.com) or Yi-Feng Yang (yyf627@163.com)

Supplementary Figure 1

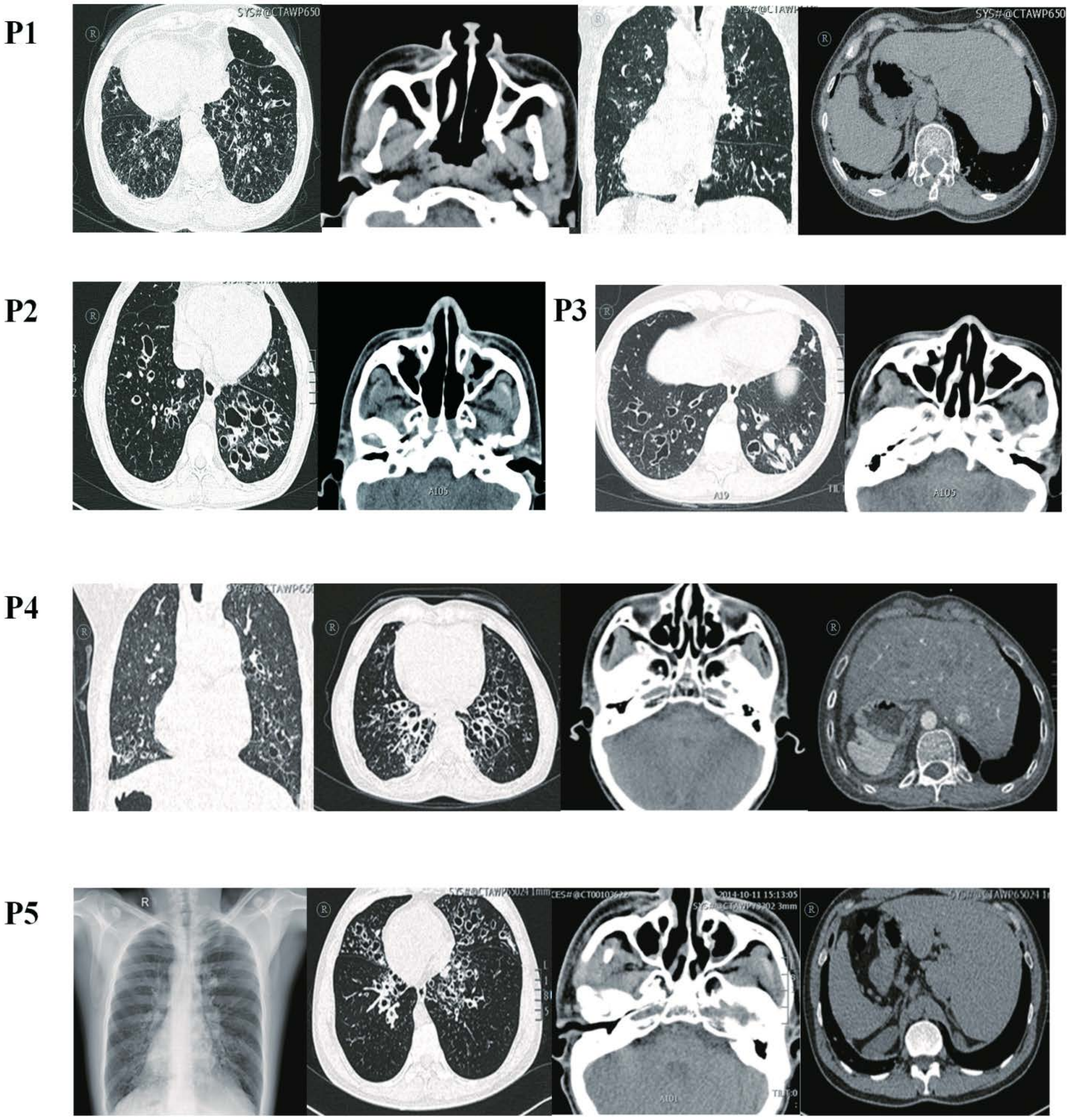

Supplementary Figure 1. Clinical features of PCD patients in our study

P1 (family1:IV-4), P2 (family2:IV-1), P3 (family2:IV-2), P4 (family3:IV-2) and P5 (family3:IV-3) represent the proband in each family, respectively. The patients presented classical clinical features including bronchiectasis, chronic rhinosinusitis or laterality defect (except for P2 and P3), as suggested by x-ray and CT scan.

Supplementary Figure 2

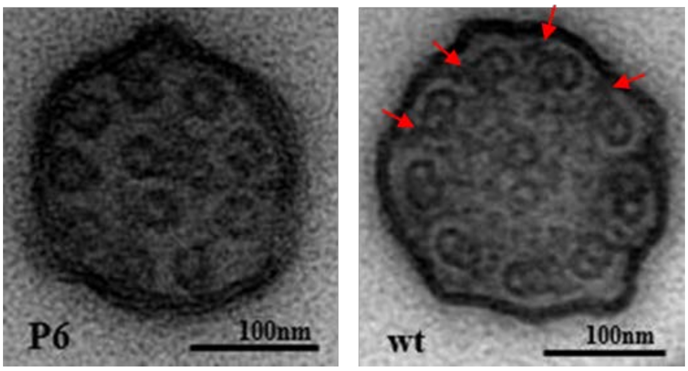

Supplementary Figure 2. Ciliary ultrastructure by electron microscope for P6

The ciliary ultrastructures of P6 and the control were shown. Arrows (in red) indicate some visible ODA structures. Due to confounding factors, the repeated results are vague and equivocal; scale bar, 100 nm

Supplementary Figure 3

Exome sequencing and filter procedure

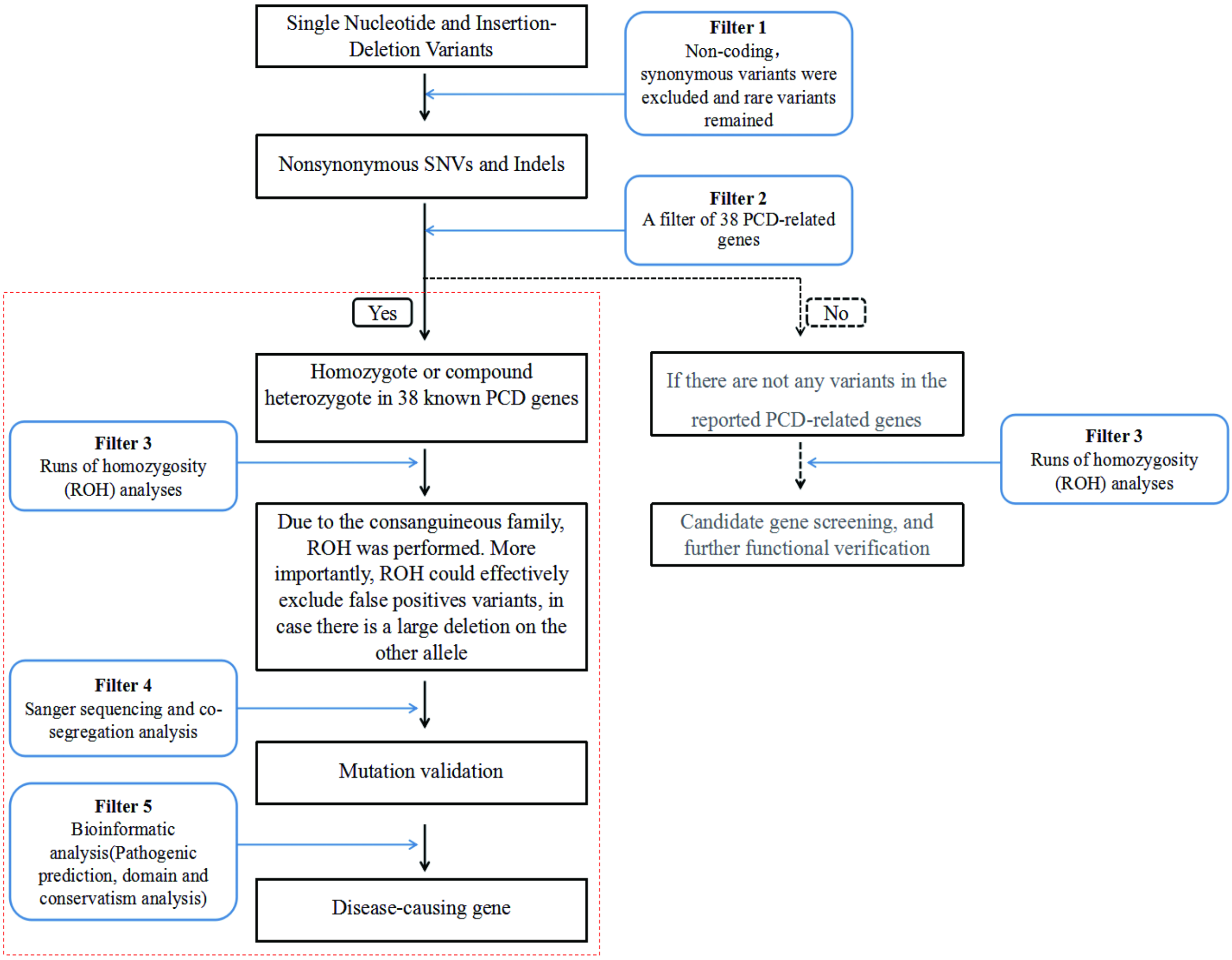

Supplementary Figure 3. Exome sequencing and filter procedure

Schematic representation of the filter strategies employed in our study.

Supplementary Figure 4

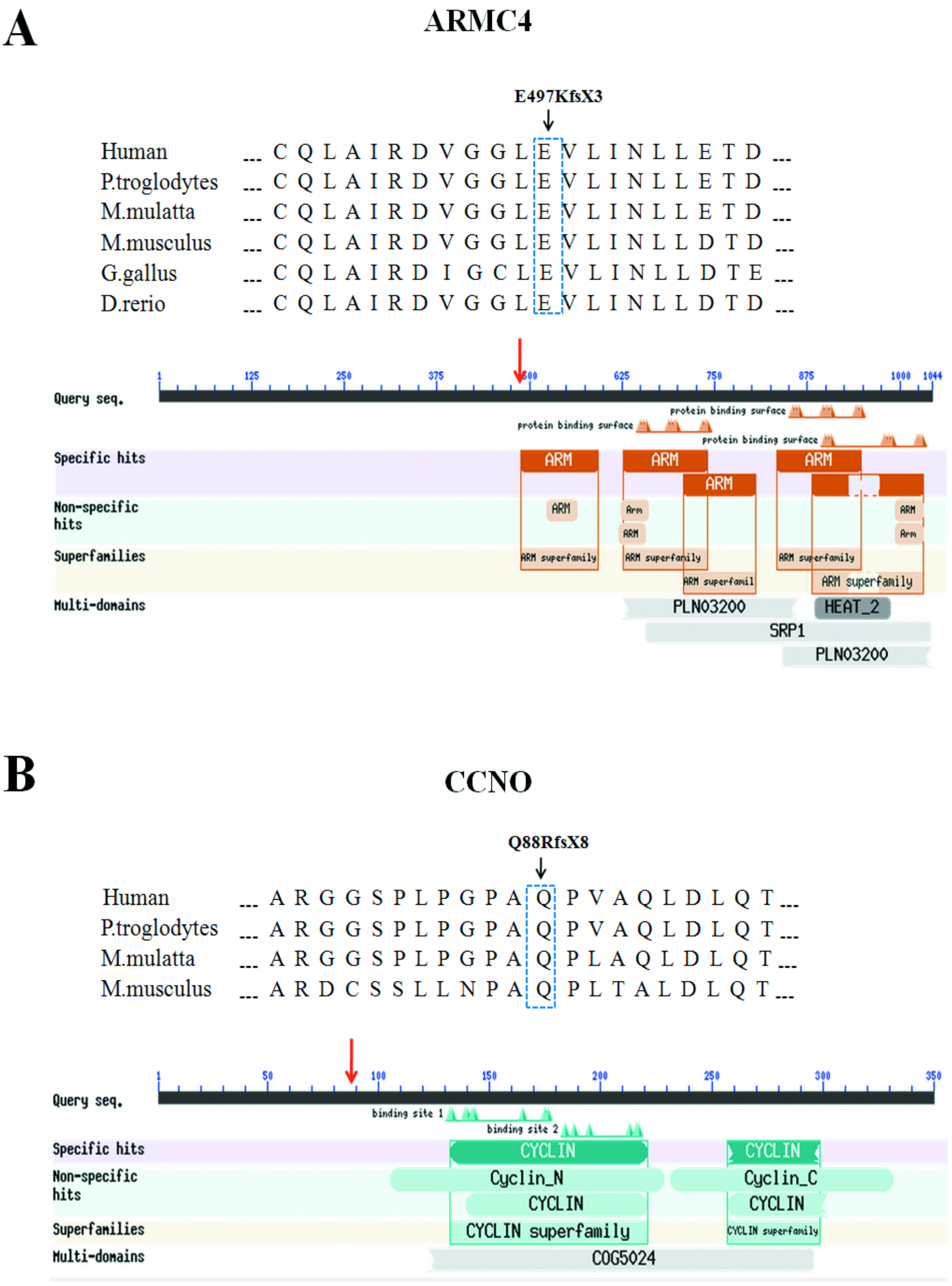

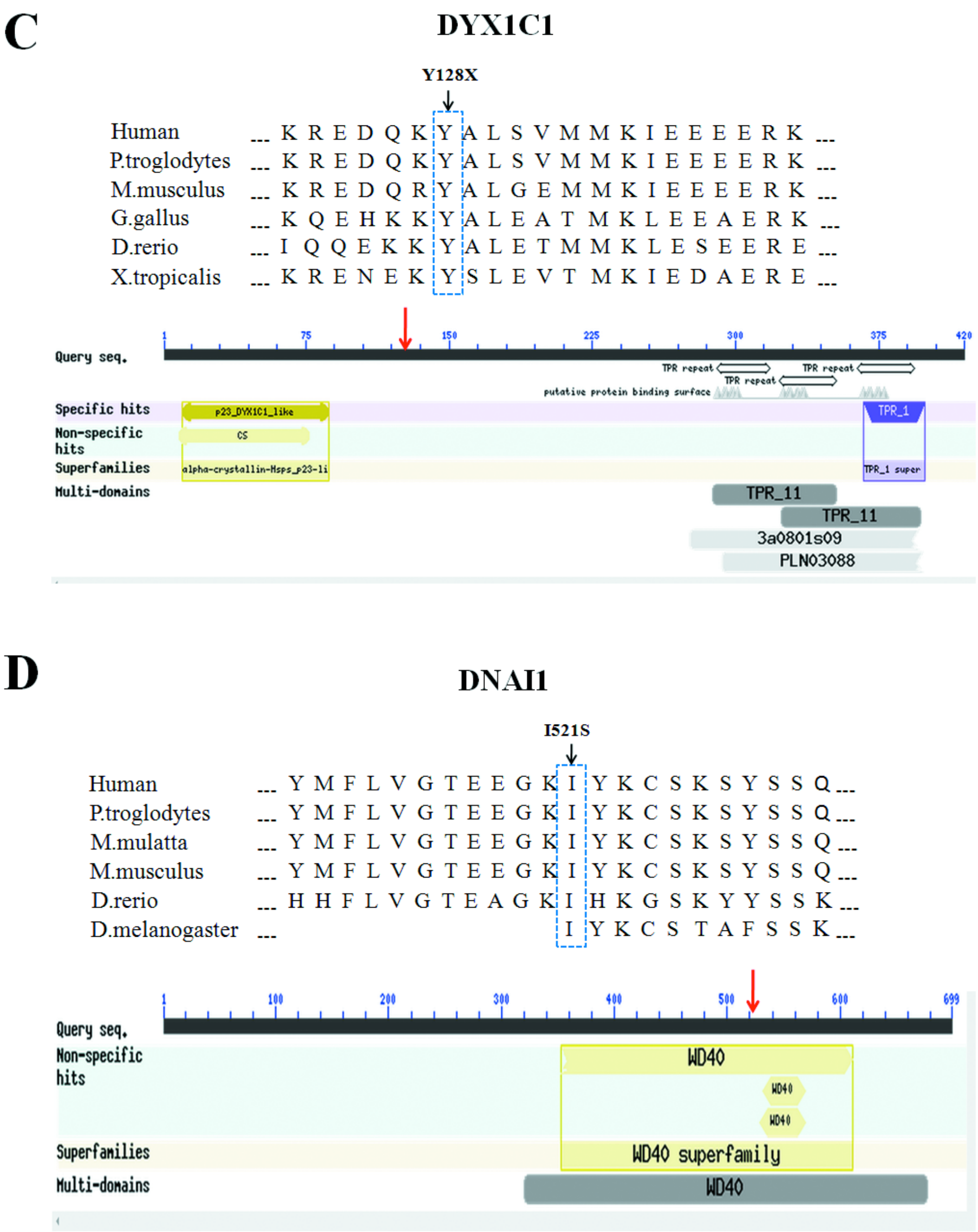

Supplementary Figure 4. The identification of conservatism and mutation domain

(A) The glutamic acid at position 497 in ARMC4 protein was highly conserved in diverse species (from MutationTaster). This frameshift mutation (c.1488delG/p.E497Kfs*3) was located in the ARMs domain (from NCBI Conserved Domain Search) and would result in premature protein truncation (from MutationTaster). (B) Alignment of multiple CCNO protein sequences across species. The Q88 affected amino acid is located in the highly conserved amino acid region in different species (from MutationTaster).

The mutation (c.248_249insGCCCG/p.Q88Rfs*8) would result in premature CCNO protein truncation, which affects the key domain--the cyclin domain (from NCBI Conserved Domain Search). (C) The residue Tyr128 in DYX1C1 protein is highly evolutionarily conserved in diverse species (from MutationTaster). Y128* would result in premature DYX1C1 protein truncation, which affects the key domain--the TPR domain(from NCBI Conserved Domain Search). (D) The isoleucine at position 521 in DNAI1 protein highly conserved in diverse species (from MutationTaster). I521 is located in the WD40 domain(from NCBI Conserved Domain Search), which plays an important role in ciliary dysfunction.

Supplementary Figure 5

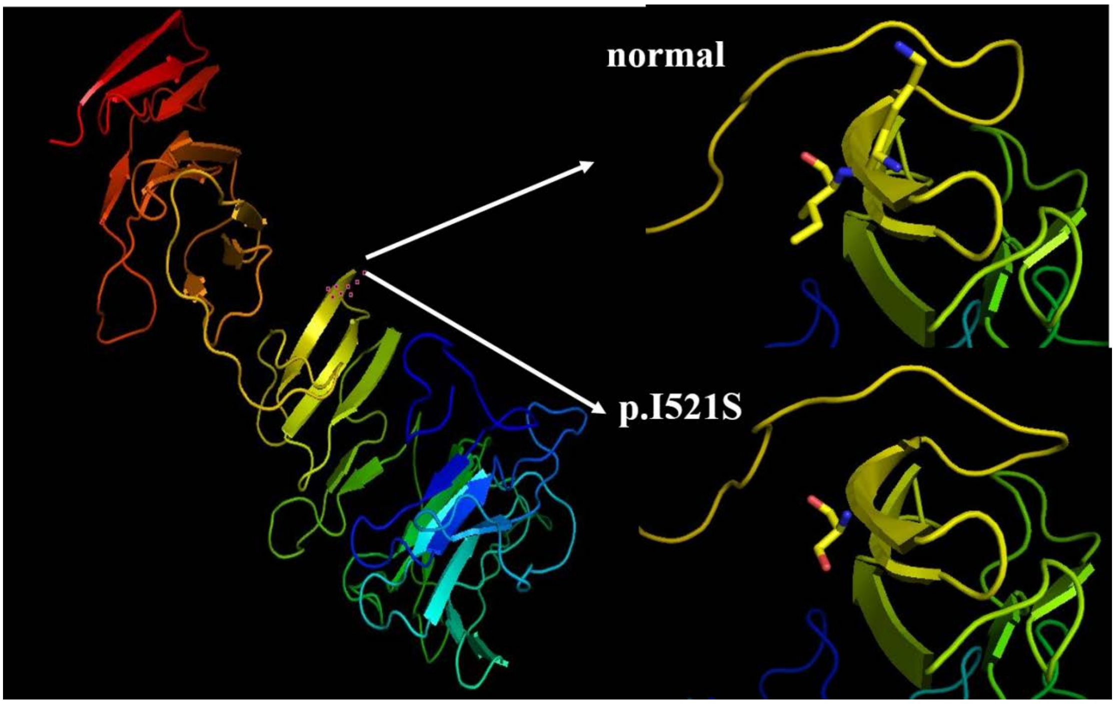

Supplementary Figure 5. Protein structure prediction of c.1562T>G/p.I521S in DNAI1

This figure shows the obvious difference between the mutant (p.I521S) and the wild type (by Swiss Model).

Supplementary Table S1: Summary of SNPs for exome captured samples and filter procedure

	Family 1	Family 2		Family 3		Family 4	
	IV-4*	IV-1*	IV-2*	IV-2*	IV-3*	IV-5*	V-1
**Exome Capture Statistics**							
Raw data(G)	7.88	5.65	4.9	8.63	8.23	5.46	6.48
Total reads (Clean reads)	52036638	43603310	37806418	66364436	63182192	41458944	49066882
Total_effective_reads	52022905	43540869	37775699	66366715	63180861	41345878	48886021
Effective_sequences_on_target(Mb)	4780.02	3433.06	2996.41	5563.37	5316.98	3365.95	4071.21
Fraction_of_effective_bases_on_target	61.8%	63.3%	63.7%	67.4%	67.6%	65.5%	67.0%
Average_sequencing_depth_on_target	94.86	68.13	59.46	110.40	105.52	66.80	80.79
Base_covered_on_target	50270523	50305471	50235525	50304372	50254766	50233534	50293815
Coverage_of_target_region	99.8%	99.8%	99.7%	99.8%	99.7%	99.7%	99.8%
Fraction of target covered＞=4×（%）	99.6%	99.4%	99.2%	99.6%	99.4%	99.2%	99.4%
Fraction of target covered＞=10×（%）	99.0%	97.8%	97.0%	98.8%	98.6%	97.3%	97.8%
Fraction of target covered＞=20×（%）	96.8%	92.1%	89.8%	96.6%	96.3%	91.2%	93.1%
**SNPs for exome capture**							
**Total number of SNVs**	105171	36253	36168	36385	36156	35866	36511
Missense	8693	7885	7860	7804	7838	7760	7945
Nonsense	61	50	55	51	55	41	48
Splice site	2124	1562	1578	1605	1574	1549	1566
Synonymous-coding	9980	9163	9166	9159	9147	9104	9215
Hom	46434	15680	15544	15597	15610	15669	15288
Het	58737	20573	20624	20788	20546	20197	21223
**Total number of indels**	12960	2291	2326	2446	2460	2368	2412
frameshift_deletion	79	65	65	65	67	74	67
frameshift_insertion	54	39	46	43	44	45	43
nonframeshift_deletion	165	101	105	89	87	87	92
nonframeshift_insertion	158	95	100	93	105	102	106
stopgain	6	3	2	5	5	3	4
Hom	6858	1289	1275	1348	1368	1332	1315
Het	6102	1002	1051	1098	1092	1036	1097

Filter procedure				
Homozygote in PCD-related genes	1	1	1	1
Compound heterozygote in PCD-related genes	0	0	0	0
Runs of homozygosity (ROH)	1	1	1	1
Bioinformatics analysis and co-segregating	1	1	1	1

*Index patient.

Supplementary Table S2. 40 PCD-related genes for filter[1-8]

PCD genes	Chromosomal location	Ciliary structural defect	OMIM
DNAH5	5p15.2	ODA defect	603335
DNAI1	9p21-p13	ODA defect	604366
DNAI2	17q25	ODA defect	605483
DNAL1	14q24.3	ODA defect	614017
TXNDC3 (NME8)	7p14-p13	Partial ODA defect	607421
CCDC114	19q13.32	ODA defect	615038
CCDC151	19q13.32	ODA defect	615956
ARMC4	10p12.1-p11.23	ODA defect	615408
DNAAF1 (LRRC50)	16q24.1	ODA and IDA defect	613190
DNAAF2 (KTU)	14q21.3	ODA and IDA defect	612517
DNAAF3 (C19ORF51)	19q13.42	ODA and IDA defect	614566
CCDC103	17q21.31	ODA and IDA defect	614677
C21orf59	21q22.1	ODA and IDA defect	615494
DYX1C1	15q21.3	ODA and IDA defect	608706
LRRC6	8q24	ODA and IDA defect	614930
HEATR2	7p22.3	ODA and IDA defect	614864
SPAG1	8q22	ODA and IDA defect	603395
ZMYND10	3p21.31	ODA and IDA defect	607070
CCDC39	3q26.33	IDA defect + microtubular disorganization	613798
CCDC40	17q25.3	IDA defect + microtubular disorganization	613799
CCDC65 (DRC2)	12q13.12	Mostly normal, CA defects in small proportion of cilia	611088

CCDC164 (DRC1)	2p23.3	Nexin (N-DRC) link missing; axonemal disorganization in small proportion of cilia	615288
RSPH1	21q22.3	Mostly normal, CA defects in small proportion of cilia	609314
RSPH4A	6q22.1	Mostly normal, CA defects in small proportion of cilia	612649
RSPH9	6p21.1	Mostly normal, CA defects in small proportion of cilia	612648
HYDIN	16q22.2	Normal, very occasionally CA defects	610812
DNAH11	7p21	Normal	603339
CCNO	5q11.2	ciliary aplasia/oligoplasia	607752
MCIDAS	5q11.2	ciliary aplasia/oligoplasia	614086
DNAH8	6p21.1	ODA defect	603337
RPGR	Xp21.1	Mixed	312610
OFD1	Xq22	Normal	300170
RSPH3	6q25.3	Central pair defect or normal	616481
GAS8	16q24.3	N-DRC defect	605178
DNAH6	2p11.2	Central pair defects	603336
TTC25	17q21.2	ODA defect	617092
DNAJB13	11q13.4	Central microtubules defect	610263
PIH1D3	Xq22.3	ODA and IDA defect	300991
CCDC11	18q21.1	No morphologic defects	614759
STK36	2q35	RS and CA defect	607652

ODA: outer dynein arm; IDA: inner dynein arm; CA: central apparatus; N-DRC: nexin-dynein regulatory complex; RS: radial spoke; NA: not available

References:

[1] Lobo, J., Zariwala, M. A. & Noone, P. G., Primary ciliary dyskinesia. Semin Respir Crit Care Med **36** 169 (2015).

[2] Shapiro, A. J. *et al.*, Diagnosis, monitoring, and treatment of primary ciliary dyskinesia: PCD foundation consensus recommendations based on state of the art review. Pediatr Pulmonol **51** 115 (2016).

[3] Olbrich, H. *et al.*, Loss-of-Function GAS8 Mutations Cause Primary Ciliary Dyskinesia and Disrupt the Nexin-Dynein Regulatory Complex. AM J HUM GENET **97** 546 (2015).

[4] Li, Y. *et al.*, DNAH6 and Its Interactions with PCD Genes in Heterotaxy and Primary Ciliary Dyskinesia. PLOS GENET **12** e1005821 (2016).

5 Wallmeier, J. *et al.*, TTC25 Deficiency Results in Defects of the Outer Dynein Arm Docking Machinery and Primary Ciliary Dyskinesia with Left-Right Body Asymmetry Randomization. AM J HUM GENET **99** 460 (2016).
6 El, K. E. *et al.*, Mutations in DNAJB13, Encoding an HSP40 Family Member, Cause Primary Ciliary Dyskinesia and Male Infertility. AM J HUM GENET **99** 489 (2016).
7 Paff, T. *et al.*, Mutations in PIH1D3 Cause X-Linked Primary Ciliary Dyskinesia with Outer and Inner Dynein Arm Defects. AM J HUM GENET **100** 160 (2017).
8 Edelbusch, C. et al., Mutation of serine/threonine protein kinase 36 (STK36) causes primary ciliary dyskinesia with a central pair defect. HUM MUTAT (2017).

Supplementary Table S3: Primers for PCR validation

chromosome	Site	CDS	Gene Name	Primer F(5'-3')	Primer R(5'-3')
Chr10	28233788	c.1488delG	*ARMC4*	TTCTAGCCCACTCAGAGACA	TCAGCAGGTCATCAGCAATAA
Chr5	54529089	c.248_249insGCCCG	*CCNO*	GGACAACGACCAGAACCTT	CTCTACCAGCACCTCACTTG
Chr15	55783341	c.384delC	*DYX1C1*	CCTTGTGAAGTTGGGTACAAAG	AGCAGCTGGAACTCACTATC
Chr9	34513182	c.1562T＞G	*DNAI1*	GCCCTAGTTCAGTCTGTCCTG	CATTTAGCCCTTATTCCTATCAC

Supplementary Table S4. The coverage of the 40 known PCD genes by the exome capture kit in PCD patients

GeneName	P1	P2	P3	P4	P5	P6
MCIDAS	1	1	1	1	1	1
CCNO	1	1	1	1	1	1
DNAAF3(C19ORF51)	1	1	1	1	1	1
DYX1C1	1	1	1	1	1	1
DNAI1	1	1	1	1	1	1
RPGR	1	1	1	1	1	1
CCDC151	1	1	1	1	1	1
DNAI2	1	1	1	1	1	1
RSPH1	1	1	1	1	1	1
DNAL1	1	1	1	1	1	1
CCDC40	1	1	1	1	1	1
DNAAF1(LRRC50)	1	1	1	1	1	1
GAS8	1	1	1	1	0.994658	1
DNAH8	1	1	1	1	1	0.999406
HYDIN	1	1	1	1	1	1
RSPH4A	1	0.984203	1	1	1	1
LRRC6	1	1	1	1	1	1
SPAG1	1	1	1	1	1	0.997352
RSPH3	1	1	1	1	1	1
DNAH6	1	1	1	1	1	1
CCDC39	1	1	1	1	1	1
HEATR2	1	0.995946	1	1	0.979189	0.99027
CCDC164(DRC1)	1	1	1	1	1	1
C21orf59	1	1	1	1	1	1
DNAH5	1	1	1	1	1	1
OFD1	1	1	1	1	1	1
TXNDC3(NME8)	1	1	1	1	1	1

CCDC103	1	1	1	1	1	1
RSPH9	1	1	1	1	1	1
DNAH11	1	1	1	1	1	1
CCDC114	1	1	1	1	1	1
CCDC65(DRC2)	1	1	1	1	1	1
DNAAF2(KTU)	1	1	1	1	1	1
ZMYND10	1	1	1	1	1	1
ARMC4	0.978439	0.992735	0.993204	1	0.998125	1
TTC25	1	0.998691	0.994108	0.990507	0.997709	0.997381
DNAJB13	1	1	1	1	1	1
PIH1D3	1	1	1	1	1	1
CCDC11	1	1	1	1	1	1
STK36	1	1	1	1	1	1

Supplementary Table S5. Frequencies of the identified variants in database

	dbSNP138	1000G	ESP6500	ExAC	ClinVar
ARMC4(c.1488delG/p.E497Kfs*3)	0	0	0	0	0
CCNO(c.248_249ins GCCCG/p.Q88Rfs*8)	0	0	0	0.0003	0
DYX1C1(c.384delC/p.Y128*)	0	0	0	0	0
DNAI1(c.1562T>G/p.I521S)	rs568312736	0.0002	0	0	0

dbSNP138: The Single Nucleotide Polymorphism database 138; 1000G: 1000 Genomes Project 2014 oct; ESP6500: NHLBI-ESP 6500 exomes database; ExAC: The Exome Aggregation Consortium
